# Catalysis-dependent and redundant roles of Dma1 and Dma2 in maintenance of genome stability in *Saccharomyces cerevisiae*

**DOI:** 10.1016/j.jbc.2021.100721

**Published:** 2021-04-29

**Authors:** Andrew R. Yoblinski, Seoyoung Chung, Sophie B. Robinson, Kaitlyn E. Forester, Brian D. Strahl, Raghuvar Dronamraju

**Affiliations:** 1 Department of Biochemistry & Biophysics, University of North Carolina School of Medicine, Chapel Hill, North Carolina, USA; 2 Lineberger Comprehensive Cancer Center, University of North Carolina School of Medicine, Chapel Hill, North Carolina, USA; 3 Curriculum in Genetics and Molecular Biology, University of North Carolina at Chapel Hill, Chapel Hill, North Carolina, USA

**Keywords:** Dma1, Dma2, homologous recombination, genome stability, DNA end resection, histones, DNA repair, yeast, ChIP-qPCR, chromatin immunoprecipitation–quantitative PCR, Co-IP, coimmunoprecipitation, DDR, DNA damage response, DSBs, DNA double-strand breaks, FHA, forkhead-associated, *GAL*, galactose, HA, hemagglutinin, HO, homothallic, HR, homologous recombination, NHEJ, nonhomologous end joining, RING, Really Interesting New Gene, SC, synthetic complete, YPD, yeast extract–peptone–dextrose

## Abstract

DNA double-strand breaks (DSBs) are among the deleterious lesions that are both endogenous and exogenous in origin and are repaired by nonhomologous end joining or homologous recombination. However, the molecular mechanisms responsible for maintaining genome stability remain incompletely understood. Here, we investigate the role of two E3 ligases, Dma1 and Dma2 (homologs of human RNF8), in the maintenance of genome stability in budding yeast. Using yeast spotting assays, chromatin immunoprecipitation and plasmid and chromosomal repair assays, we establish that Dma1 and Dma2 act in a redundant and a catalysis-dependent manner in the maintenance of genome stability, as well as localize to transcribed regions of the genome and increase in abundance upon phleomycin treatment. In addition, Dma1 and Dma2 are required for the normal kinetics of histone H4 acetylation under DNA damage conditions, genetically interact with *RAD9* and *SAE2*, and are in a complex with Rad53 and histones. Taken together, our results demonstrate the requirement of Dma1 and Dma2 in regulating DNA repair pathway choice, preferentially affecting homologous recombination over nonhomologous end joining, and open up the possibility of using these candidates in manipulating the repair pathways toward precision genome editing.

DNA double-strand breaks (DSBs) are among the most deleterious lesions in the genome. Accordingly, organisms have developed various mechanisms by which to repair this kind of damage (1). Eukaryotes typically use one of two primary DSB repair pathways, homologous recombination (HR) or nonhomologous end joining (NHEJ), although alternative repair pathways also exist (2, 3, 4). Mammalian cells tend to favor the NHEJ pathway, although HR is also used during the S-G2 phases of the cell cycle (5, 6, 7). However, in budding yeast, DNA repair occurs preferentially through HR (8, 9), a process dependent upon Rad52 (10, 11, 12, 13). The DNA damage response (DDR), which is activated upon induction of DNA damage and relatively well characterized in both yeast and mammals (14), involves the activation of ataxia telangiectasia mutated (ATM) and ATM and RAD3-related kinases and binding of the Mre11-Rad50-Xrs2/Nbs1 complex to the ends of DSBs to initiate the process of DNA repair (2, 3, 15, 16). Based on the pathway chosen, either KU70/80 are recruited to the ends to perform NHEJ (17, 18, 19), or the Mre11-Rad50-Xrs2/Nbs1 complex initiates the process of end resection, generating long ssDNA that are coated by replication protein A, which is then exchanged for Rad51, completing the process of HR upon identification of a homologous template (20, 21, 22, 23).

The earliest histone post-translational modification to be induced upon DNA damage is the phosphorylation of variant histone H2A.X (mediated by the kinases ATM/ATM and RAD3-related) (24, 25, 26) and histone H4ser1 (mediated by casein kinase II) (27, 28, 29). In addition to phosphorylation, histone acetylation and methylation have been shown to have an important role in DNA damage repair by recruiting various repair complexes (30, 31, 32, 33). Phosphorylation of H2A.X acts as a docking site for the mediator of DNA damage checkpoint 1 in metazoan cells (34, 35, 36). The N-terminal forkhead-associated (FHA) domain of the mediator of DNA damage checkpoint 1 is extensively phosphorylated and plays an important role in the recruitment of the E3 ligases RNF8/RNF168 (mammalian homologs of Dma1 and Dma2), *via* their FHA domains, resulting in ubiquitylation of H2A.X (37, 38, 39). This ubiquitylation is required for the recruitment of repair proteins such as 53BP1 (Rad9 in yeast), Rad51 (14, 38, 40, 41), and CHD4 for chromatin relaxation (42). The importance of RNF8 is underscored by the increase in spontaneous tumorigenesis in mice lacking RNF8 and certain human DNA repair deficiency disorders such as the RIDDLE syndrome (43, 44). The budding yeast homologs of RNF8, Dma1 and Dma2, are similar with respect to their domain organization (Fig. 1*A*) (45). The N-terminal FHA domain of Dma2 has been shown to interact with DDR proteins such as Rad9 and Sae2 in a DNA damage–dependent manner (46). The E3 ubiquitin ligase domains of Dma1 and Dma2 are very similar to the highly conserved Really Interesting New Gene (RING) domain (47). Dma1 and Dma2 are 58% identical and have been previously characterized as functionally redundant (47). Furthermore, Dma1 and Dma2 have been implicated in cell cycle control by regulating septin dynamics (48), spindle position checkpoint (47), regulation of protein kinase Swe1 (49), and DNA replication control (50).

**Figure 1:**
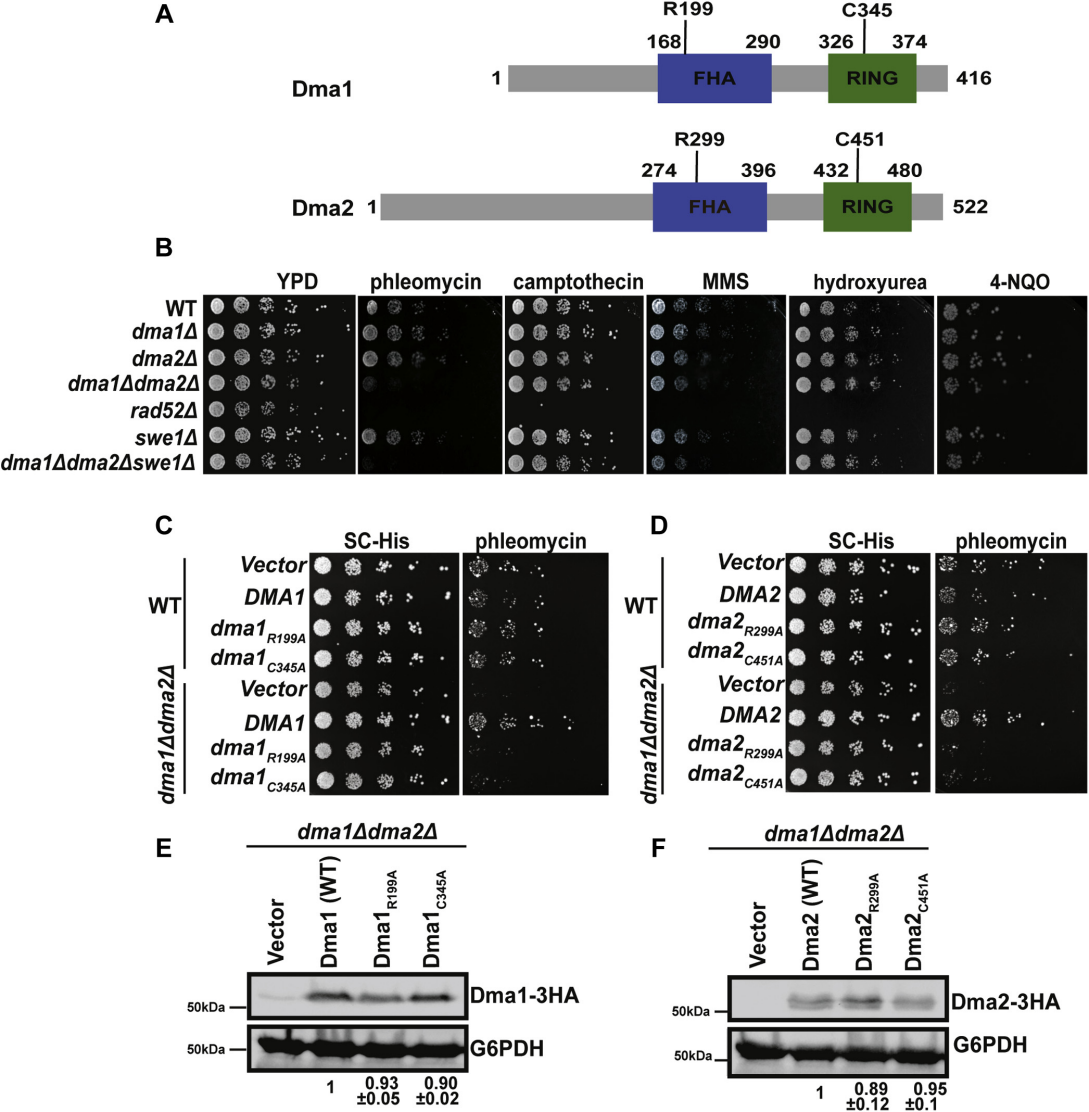
**Dma1 and Dma2 are required for resistance to phleomycin in a catalysis-dependent manner.***A*, the schematic of structure and functional domains of Dma1 and Dma2 proteins. Dma1 and Dma2 both contain an FHA (binding) domain and a RING (catalytic) domain. *B*, spotting of WT and *dma1Δdma2Δ* strains on several genotoxic drug plates, including YPD (2% dextrose), phleomycin (2.5 μg/ml), camptothecin (5 μg/ml), MMS (0.0125%), hydroxyurea (200 mM), 4-nitroquinoline 1-oxide (4NQO) (5 μg/ml). *C*, the spotting assay showing the catalysis and FHA domain dependence of Dma1. *D*, Dma2 for survival. Yeast cells were transformed with WT and mutant DMA2 plasmids as described in Experimental procedures and spotted on synthetic complete media lacking histidine (SC-HIS) and phleomycin (10 μg/ml). *Panels E* and *F* are the immunoblots showing the levels of HA-tagged Dma1 and Dma2 in the yeast strains. Immunoblots were performed as described in Experimental procedures. FHA, forkhead-associated; HA, hemagglutinin; MMS, methyl methanesulfonate; RING, Really Interesting New Gene; YPD, yeast extract–peptone–dextrose.

Although these homologs of RNF8 are present in budding yeast, a role for Dma1 and Dma2 in the maintenance of genome stability in budding yeast has not been addressed. Given the homology between Dma1 and Dma2 and RNF8, we sought to investigate the role of Dma1 and Dma2 in the maintenance of genome stability. We show that Dma1 and Dma2 function in a redundant manner for phleomycin resistance but not to other genotoxic agents. Furthermore, the resistance to phleomycin is dependent on both the E3 ligase activity of the RING domain and the phospho-substrate binding activity of the FHA domain. Dma1 and Dma2 localize to gene bodies in the absence of DNA damage, suggesting a role in genome maintenance during transcription. The levels of Dma1 and Dma2 on gene bodies increase dramatically upon phleomycin treatment in a manner similar to phosphorylation of histone H2Aser129. Coimmunoprecipitation (Co-IP) studies show that both Dma1 and Dma2 interact with histones and Rad53. Furthermore, Dma1 and Dma2 are required for proper induction of H4 acetylation upon phleomycin treatment. Genetically, *DMA1* and *DMA2* individually are epistatic with *RAD9* upon phleomycin treatment, and they display a synthetic sickness when both are deleted together with *SAE2*. Finally, plasmid-based and chromosomal repair assays show that Dma1 and Dma2 are required for HR fidelity and likely participate in repair pathway choice, favoring HR over NHEJ. Overall, our studies identify novel candidates involved in genome maintenance with broad implications in genome editing and cancer biology.

## Results

### Redundant and catalysis-dependent roles of Dma1 and Dma2 in DSB survival

Previous studies show that Dma1 and Dma2 are required for cell cycle control *via* regulating the stability of the *Saccharomyces cerevisiae* Swe1 (Wee1) protein (49). Given cell cycle control and the regulation of DNA damage repair are intimately connected, we asked if Dma1 and Dma2 might also be required to maintain genome stability. To test their requirement in this function, we constructed deletion strains of *DMA1* and *DMA2* and spotted them as serial dilutions on plates containing various genotoxic agents. These spotting assays revealed that deletion of either *DMA1* or *DMA2* (*dma1Δ* and *dma2Δ*, respectively) alone did not display any slow growth or lethality when exposed to several genotoxic drugs (Fig. 1*B*). However, a combined deletion of both *DMA1* and *DMA2* together (*dma1Δdma2Δ*) displayed extreme sensitivity to phleomycin (Fig. 1*B*). Interestingly, *dma1Δdma2Δ* cells were not sensitive to any other genotoxic agents tested, including camptothecin, methyl methanesulfonate, hydroxyurea, and 4-nitroquinoline 1-oxide at indicated concentrations (Fig. 1*B*). Given phleomycin causes global DSBs by an unknown mechanism, we also tested the effects of deletion of *DMA1* and *DMA2* on plates containing an established radiomimetic drug Zeocin. As shown in Figure S1, and in a manner similar to phleomycin, a combined deletion of both *DMA1* and *DMA2* together displayed extreme sensitivity to Zeocin, confirming that Dma1 and Dma2 are specifically required for the repair of DSBs.

During replication stress, Swe1 phosphorylates Tyr 15 on cyclin-dependent kinase Cdc28, thereby preventing cells from entering into mitosis in the presence of replication defects or DNA damage (51, 52). Given that Dma1 and Dma2 regulate the degradation of Swe1, we asked if stabilization of Swe1 in the absence of Dma1 and Dma2 was responsible for the observed DNA damage phenotype. To this end, we created a yeast strain lacking *DMA1*, *DMA2*, and *SWE1*. We tested the sensitivity of the triple mutant to several genotoxic agents as described above. As shown in Figure 1*B*, we did not observe any differences in the sensitivity of the double (*dma1Δdma2Δ*) and the triple mutants (*dma1Δdma2Δswe1Δ*) to phleomycin, suggesting that stabilization of Swe1 in the absence of Dma1 or Dma2 does not contribute to the phleomycin sensitivity.

Dma1 and Dma2 are E3 ubiquitin ligases that have been shown to target various substrates, including Swe1 (49). To determine domain dependence of phleomycin sensitivity observed in *dma1Δdma2Δ* cells, we cloned full-length *DMA1* and *DMA2* into the pRS313 vector and generated single amino acid point mutations in both the FHA domains (R199A in Dma1 and R299A in Dma2) and RING domains (C345A in Dma1 and C451A in Dma2) of each protein (Fig. 1*A*). These plasmids were then introduced into the *dma1Δdma2Δ* strain by standard transformation procedures. As shown in Figure 1, *C* and *D*, respectively, the Dma1 and Dma2 point mutants displayed a phleomycin sensitivity that phenocopied the *dma1Δdma2Δ* strain, both confirming their redundant nature and demonstrating FHA and RING domain dependence for phleomycin resistance. Furthermore, as shown in Figure 1, *E* and *F*, immunoblotting revealed that the WT and mutant hemagglutinin (HA)-Dma1 and HA-Dma2 proteins were expressed at similar levels, demonstrating that the phleomycin sensitivity of the FHA and the RING point mutants was not due to stability defects of mutant proteins. Taken together, these results show that both the catalytic activity and the phospho-substrate binding ability of Dma1 and Dma2 are required for survival upon global DSB induction.

### Kinetics of Dma1 and Dma2 upon DNA damage

The first step in the process of DDR signaling is the phosphorylation of histone H2Aser129 by Mec1/Tel1, which is required for the recognition of the lesion and the subsequent signaling cascade (26). To determine if the phleomycin sensitivity of *dma1Δdma2Δ* strain might be due to defects in DDR induction kinetics, we performed chromatin immunoprecipitation followed by quantitative PCR (qPCR) (ChIP-qPCR) upon phleomycin treatment at various time points (0 min, 15 min, and 30 min) to study the induction of H2A phosphorylation on ser129. WT and *dma1Δdma2Δ* strains were grown to the log phase and treated with 250 μg/ml of phleomycin, and then samples were collected and fixed according to standard ChIP protocols. Given that phleomycin induces DNA breaks throughout the genome, we used long and highly expressed genes (*PMA1*, *TDH3*, and *NRD1*) and a transcriptionally silent region such as the *MAT* locus as candidates to assess the localization of histones and Dma1 and Dma2. As shown in Figure S2, we observed an increase in the phosphorylation of histone H2A at Serine129 within the first 15 min of phleomycin treatments. However, we did not observe a significant difference in the phospho-H2A induction kinetics at the studied loci in the *dma1Δdma2Δ* mutant compared with the WT.

**Figure 2:**
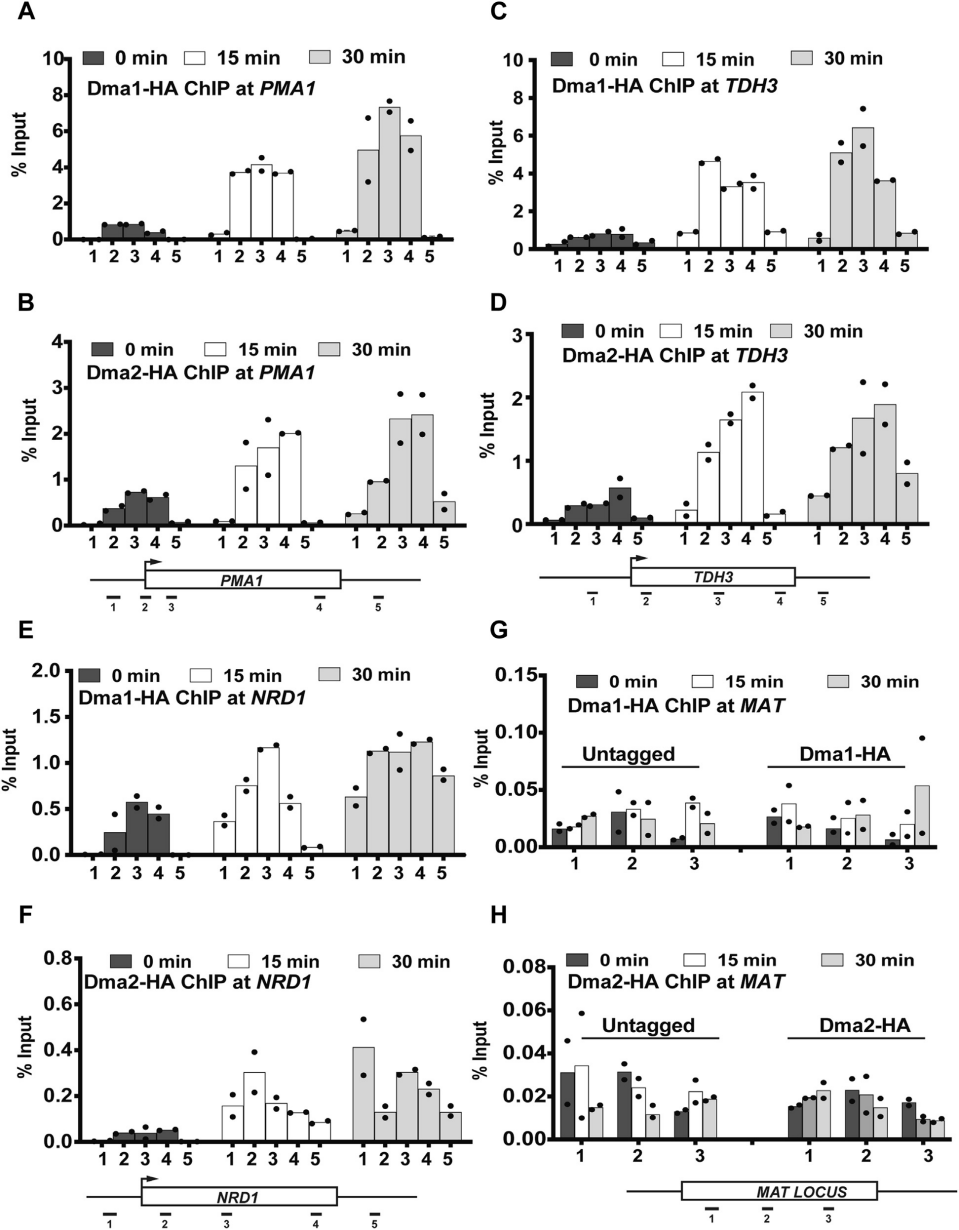
**Dma1 and Dma2 increase in abundance upon DNA damage on chromatin**. WT (untagged) and Dma1-HA and Dma2-HA tagged strains were treated with phleomycin (250 μg/ml) under asynchronously growing conditions. Cells were fixed for ChIP as described in Experimental procedures. ChIP-qPCR showing the localization of Dma1 and Dma2 on candidate loci. *A* and *B*, Dma1 and Dma2 localization on *PMA1*, respectively. *C* and *D*, Dma1 and Dma2 localization on *TDH3*, respectively. *E* and *F*, Dma1 and Dma2 localization on *NRD1*, respectively. *G* and *H*, Dma1 and Dma2 localization at the *MAT* locus, respectively. Positions of the primers on the tested-candidate loci are shown underneath the graphs. Primer sequences for the tested loci were from the study by Grzechnik *et al*. (76). ChIP-qPCR, chromatin immunoprecipitation–quantitative PCR; HA, hemagglutinin.

Next, given that the *dma1Δdma2Δ* mutant is sensitive to DSB induction by phleomycin, we asked if Dma1 and Dma2 are recruited to the damage sites upon phleomycin treatment using ChIP-qPCR at the abovementioned genes. Surprisingly, our results showed that Dma1 and Dma2 are localized to the gene bodies of *PMA1* (Fig. 2, *A* and *B*), *TDH3* (Fig. 2, *C* and *D*), and *NRD1* (Fig. 2, *E* and *F*), even in the absence of phleomycin treatment; however, upon phleomycin treatment, we observed an increase in their abundance on actively transcribed gene bodies. Importantly, we did not observe any increase in the localization of Dma1 or Dma2 at the *MAT* locus (Fig. 2, *G* and *H*), suggesting that Dma1 and Dma2 may play a role in the transcribed regions of the genome.

### FHA domain–dependent recruitment of Dma1 and Dma2 to chromatin

**Figure 3:**
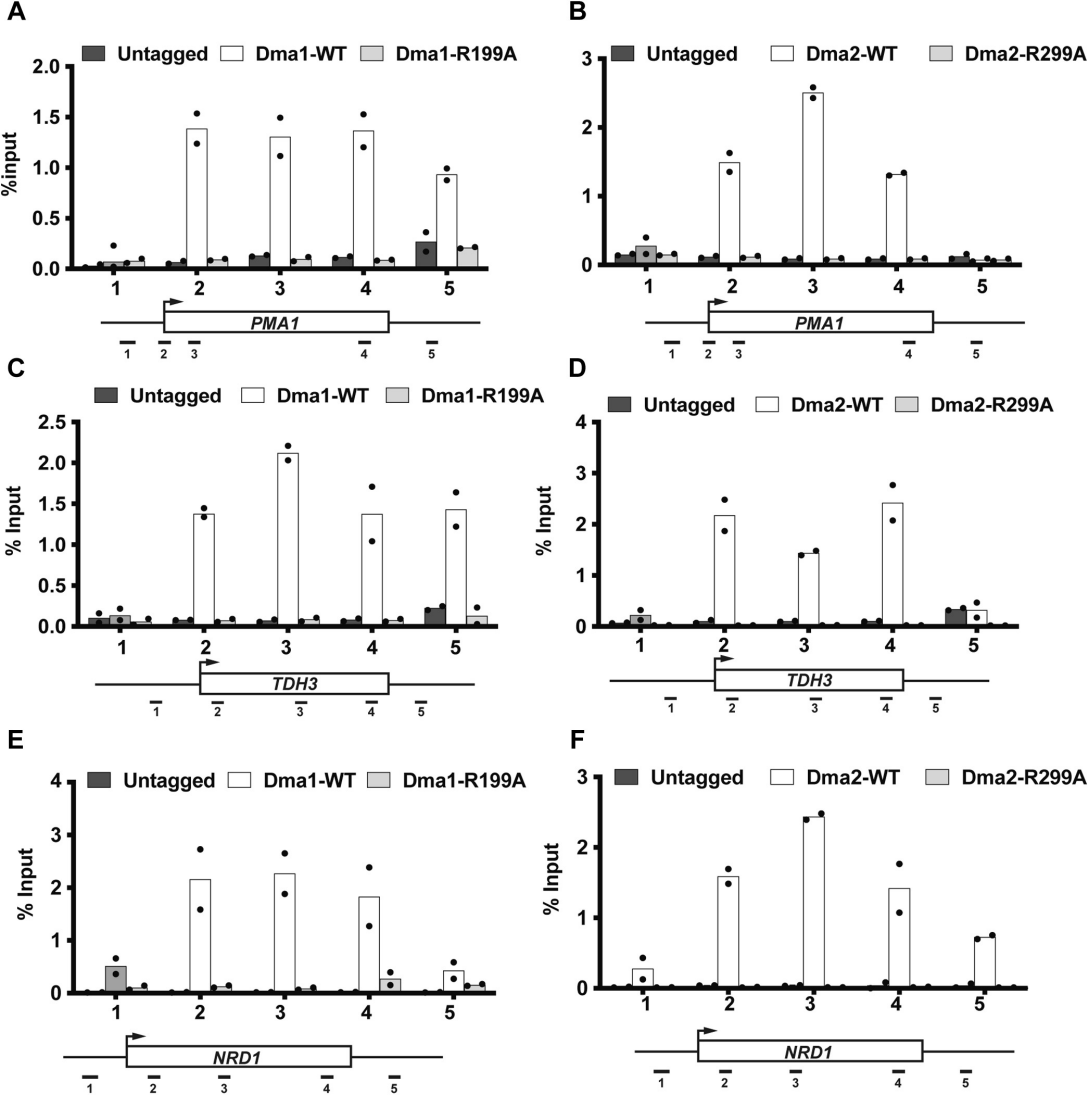
**FHA-dependent localization of Dma1 and Dma2 to gene bodies.***dma1Δdma2Δ* mutant strain transformed with PRS313 plasmid (either untagged, or with one of HA-tagged DMA1-WT, DMA2-WT, dma1-R199A, dma2-R299A) was treated with phleomycin (250 μg/ml) under asynchronously growing conditions, as described in Experimental procedures. Cells were fixed for ChIP as described in Experimental procedures. Lysates were prepared as described and immunoblotted for the HA-tagged versions of the WT and the FHA mutants of Dma1 and Dma2. *A* and *B*, Dma1 and Dma2 localization on *PMA1*, respectively. *C* and *D*, Dma1 and Dma2 localization on *TDH3*, respectively. *E* and *F*, Dma1 and Dma2 localization on *NRD1*, respectively. Positions of the primers on the tested candidate loci are shown underneath the graphs. Primer sequences for the tested loci are from the study by Grzechnik *et al*. (76). ChIP, chromatin immunoprecipitation; FHA, forkhead-associated; HA, hemagglutinin.

FHA domains are phospho-substrate–interacting domains that are involved in DNA repair processes and mediate protein interaction networks (53, 54, 55). Given that the FHA domain of both Dma1 and Dma2 are required for resistance to phleomycin, we next asked if the phenotypes correlated with the ability of the mutants to be recruited to chromatin. To this end, we performed ChIP-qPCR using WT, Dma1-R199A, and Dma2-R299A mutants to determine their recruitment to chromatin using the same candidate genes as described above. As shown in Figure 3, while WT-Dma1 and WT-Dma2 proteins localized to the gene bodies, the Dma1R199A and Dma2-R299A mutants were defective in chromatin localization at all genes examined. Thus, the inability of the mutants to be recruited to chromatin may partially explain their sensitivity to phleomycin.

### Dma1 and Dma2 interact with Rad53 and histones

**Figure 4:**
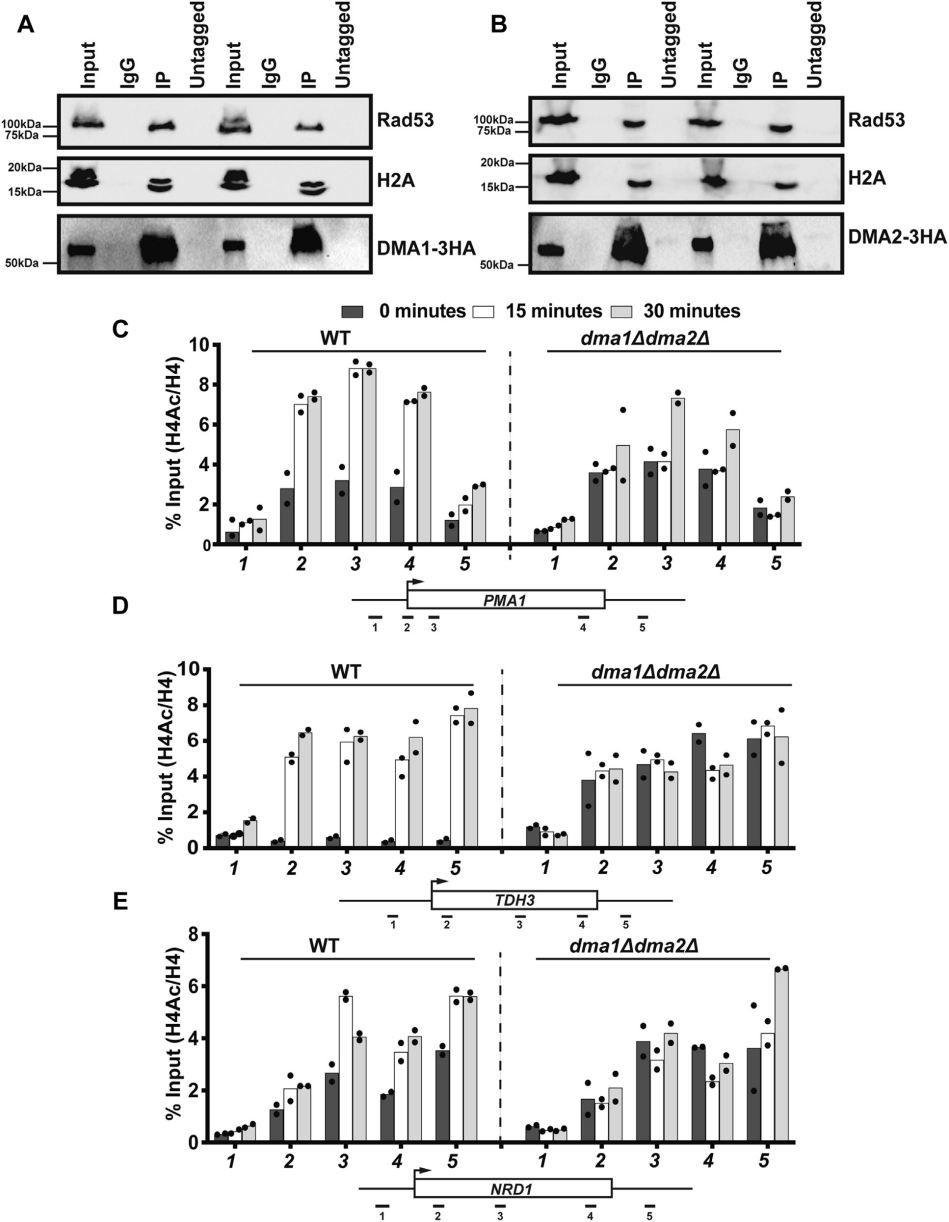
**Dma1 and Dma2 are required for chromatin structure during DNA repair.***A* and *B*, coimmunoprecipitation of 3XHA-tagged Dma1 and Dma2, respectively, was performed under conditions of either phleomycin treatment or no treatment as described in Experimental procedures and immunoblotted for various antibodies as shown. WT and *dma1Δdma2Δ* mutant strains were treated, and ChIP was performed, as described in Experimental procedures. *C–E*, H4(Ac)4 ChIP signal normalized to histone H4 on *PMA1*, *TDH3*, and *NRD1*, respectively. Positions of the primers on the tested candidate loci are shown underneath the graphs. Primer sequences for the tested loci were from the study by Grzechnik *et al*. (76). ChIP, chromatin immunoprecipitation; HA, hemagglutinin.

Given that Dma1 and Dma2 are recruited to gene bodies in an FHA domain–dependent manner, we next asked if these proteins interact with Rad53 and nucleosomes. To this end, we performed Co-IP experiments of Dma1 and Dma2 that were 3X-HA-tagged at the C terminus and probed for interacting partners. Our results showed that both Dma1 and Dma2 associate with Rad53 and H2A (Fig. 4, *A* and *B* respectively). Interestingly, our Co-IP experiments also showed that the interaction with both Rad53 and histones was independent of DNA damage, suggesting that these interactions with Dma1 and Dma2 may not be required for activation of DDR signaling, but for an unknown function. These results are consistent with the fact that we did not observe any significant defect in the induction of H2ASer129 phosphorylation in the *dma1Δdma2Δ* mutants compared with the WT (Fig. S2).

Because Dma1 and Dma2 interact with histones and localize to gene bodies, we next asked if they contribute to DNA damage–dependent changes in histone modifications other than H2A phosphorylation. DNA damage–induced acetylation of histone H4 (mediated by NuA4 complex) plays a dynamic role during the process of DNA repair (56, 57, 58, 59). Thus, we focused on this histone acetylation mark further. As shown in Figure 4, *C–E*, there was no significant difference in the levels of histone H4 acetylation in the WT and the *dma1Δdma2Δ* mutant in the absence of DNA damage at the tested loci. However, upon treatment with phleomycin, we observed a significant increase in the levels of H4Ac (as assessed by an H4 pan-acetyl antibody) in the WT cells but not in the *dma1Δdma2Δ* mutants. While correlative, these results suggest that Dma1 and Dma2 are required for proper dynamics of histone acetylation and may contribute to the recruitment of repair proteins at the sites of DNA damage.

### Dma1–Dma2 control repair pathway choice

**Figure 5:**
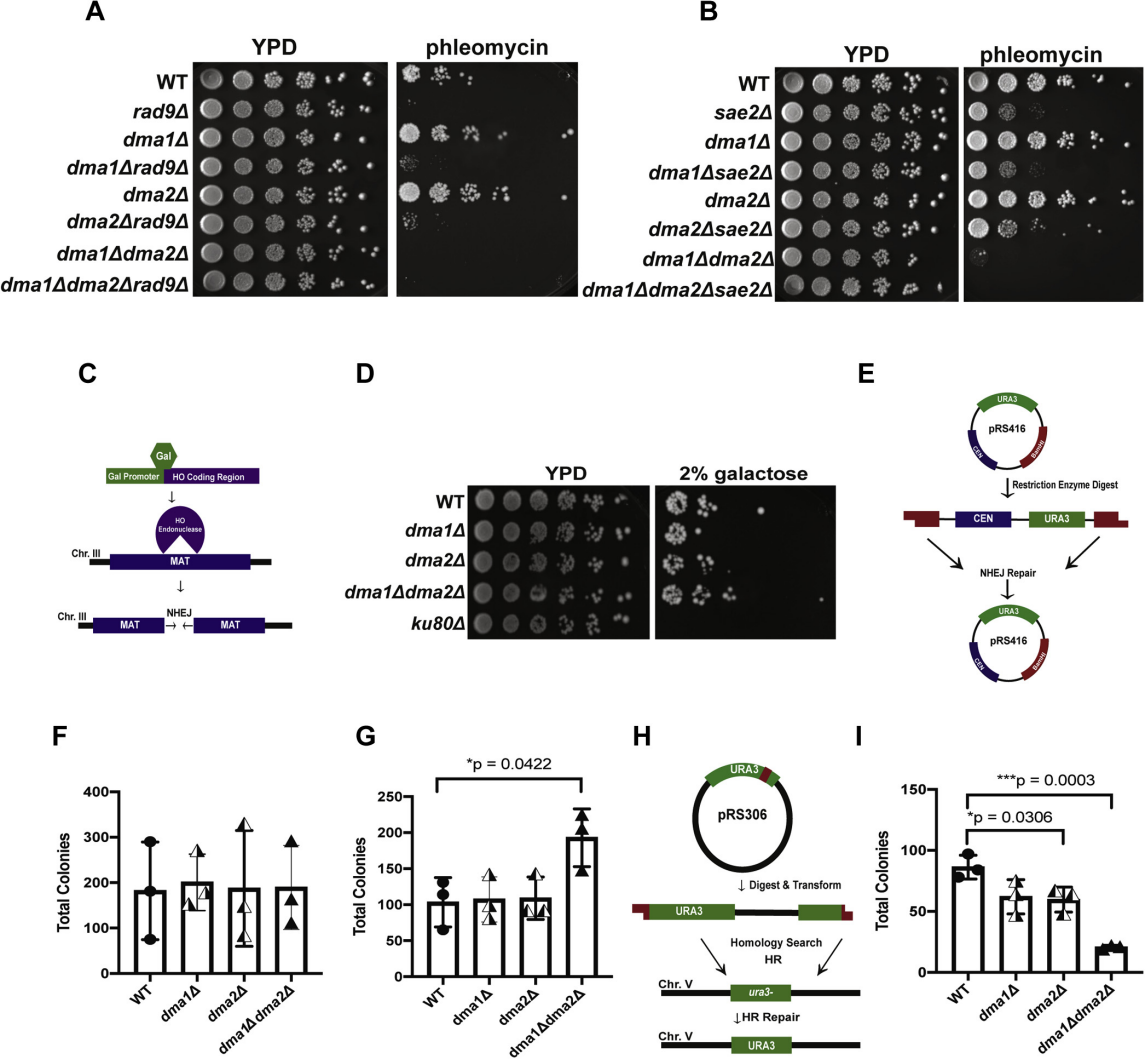
**DMA1 and DMA2 genetically interact with RAD9 and SAE2 and are required for the HR repair of DSBs.***A*, spotting assays showing the genetic interactions between DMA1 or DMA2 and RAD9 under conditions of normal growth (YPD) and upon treatment with phleomycin (2.5 μg/ml). *B*, spotting assays showing the genetic interactions between DMA1 or DMA2 and SAE2 under conditions of normal growth (YPD) and upon treatment with phleomycin (2.5 μg/ml). *C*, schematic of the galactose-inducible single DSB system. *D*, spotting of single- and the double-deletion strains on YPD (2% dextrose) and YP Gal plates (2% galactose, DSB-inducing conditions). *E*, the schematic of the plasmid-based NHEJ repair assay as detailed in Experimental procedures. *F*, the bar graph showing the plasmid transformation efficiency of pRS316 in the indicated strains. *G*, the bar graph showing the efficiency of NHEJ plasmid repair in the indicated strains. *H*, the schematic of HR repair assay through the integration of a linearized plasmid (pRS306) using the restriction enzyme StuI at the URA3 locus. I. the bar graph showing the efficiency of the integration of the plasmid at URA3 locus. All repair assays were performed in biological triplicates, and the colonies were counted manually. Alpha for all statistical analyses was 0.05, and statistically significant *p*-values for unpaired two-tailed *t* test for means are indicated by *asterisks* on graphs in all cases. DSB, DNA double-strand break; HR, homologous recombination; NHEJ, nonhomologous end joining; YPD, yeast extract–peptone–dextrose.

Given the requirement of Dma1 and Dma2 in resistance to DSB-inducing agents, we next asked if they genetically interact with repair machinery. To this end, we chose Rad9 (homolog of 53BP1, recruited in an RNF8-dependent manner) (60, 61) and Sae2 (protein involved in resection, and a protein that antagonizes the function of Rad9) (62, 63). Spotting assays revealed that *dma1Δrad9Δ* and *dma2Δrad9Δ* deletion strains did not display any growth defects under undamaged conditions (Fig. 5*A*). However, upon exposure to 10 μg/ml phleomycin, deletion of *RAD9* displayed sickness, which was partially rescued by the deletion of *DMA1* or *DMA2*. Furthermore, the *dma1Δdma2Δrad9Δ* triple mutant showed similar sensitivity on phleomycin plates compared with the *dma1Δdma2Δ* double mutant (Fig. 5*A*). Similar to RAD9, deletion of SAE2 showed sensitivity to phleomycin. Unlike RAD9, deletion of SAE2 with either DMA1 or DMA2 individually displayed a synthetic lethal interaction upon exposure to 10 μg/ml phleomycin, rather than a partial rescue (Fig. 5*B*). Interestingly, the *dma1Δdma2ΔsaeΔ* triple mutant was also synthetic sick on phleomycin compared with the *dma1Δdma2Δ* double mutant (Fig. 5*B*).

Given the genetic interaction of *DMA1* and *DMA2* with *RAD9* and *SAE2*, and given the role Sae2 is known to play in end resection, we next asked if they played any role in repair pathway choice. To investigate the role of Dma1 and Dma2 in NHEJ, we used a yeast strain (JKM179) that harbors a galactose (*GAL*)-inducible homothallic (HO) endonuclease that generates a single DSB in the *MAT* locus (Fig. 5*C*) (50). Single and double *DMA1* and *DMA2* deletions were spotted on plates containing either 2% dextrose (HO-noninducing conditions) or 2% *GAL* (HO-inducing conditions). The JKM179 strain is deleted for the homologous arms, restricting repair under 2% *GAL* to only the NHEJ pathway. To our surprise, we found that Dma1 and Dma2 were not required for the repair of this single chromosomal DSB (Fig. 5*D*) *via* NHEJ.

Using a complementary method, we assessed NHEJ repair using a plasmid-based system (64), as shown in the schematic in Figure 5*E* and detailed in Experimental procedures. As shown in Figure 5*F*, there was no significant difference in the transformation efficiency of a circular plasmid in either the single- or double-deletion mutants of *DMA1* and *DMA2* compared with the WT. We next transformed a BamHI-digested plasmid into the single and double mutants and allowed the cells to repair the break and generate colonies on plates lacking uracil. Our results showed that plasmid repair efficiency through NHEJ was not significantly different between WT and the single-deletion mutants of *DMA1* and *DMA2,* although *dma1Δdma2Δ* showed an increase (*p* = 0.0422) in efficiency of NHEJ compared with the WT (Fig. 5*G*). Overall, these data show that either Dma1 and Dma2 are dispensable in NHEJ or they potentially act to suppress NHEJ pathway selection.

We next asked if Dma1 and Dma2 are instead required for the DSB repair by HR. To this end, we used a plasmid-based assay (65), where the pRS306 *URA3*-marked plasmid was linearized using StuI (generating homologous arms for integration into the *ura−* locus converting it to *URA+*) and transformed into WT and *dma1Δdma2Δ* strains (details shown in Fig. 5*H*). As shown in Figure 5*I*, the WT and *dma1Δ* single mutant were proficient in HR repair, although the *dma2Δ* single mutant surprisingly showed reduced efficiency compared with the WT (*p* = 0.0306). Of greater interest, the *dma1Δdma2Δ* double mutant showed an even greater decrease in the efficiency of HR than the WT (*p* = 0.0003). Taken together, these data show that Dma1 and Dma2 are required for efficient HR and may play a role in regulating repair pathway choice.

## Discussion

The repair pathway adopted by a cell and the components that drive this process are of paramount importance in repairing DSBs, which is central to cancer biology and gene editing technologies such as CRISPR/Cas (66, 67, 68). In the present article, we have uncovered an important and redundant role for Dma1 and Dma2 (the budding yeast homologs of human RNF8) in the maintenance of genome stability and in pathway choice selection in DSB repair. This function of Dma1 and Dma2 requires the ubiquitin ligase activity of the RING domain, and the phospho-substrate binding activity of the FHA domain, although the precise targets of these enzymes remain elusive. Dma1 and Dma2 localize to gene bodies in the absence of DNA damage and increase in a manner similar to H2A-Ser129 phosphorylation upon phleomycin treatment. Furthermore, we show that Dma1 and Dma2 interact with Rad53 and histones and play a role in modulating histone H4 acetylation upon DNA damage. Finally, we showed that Dma1 and Dma2 are required for the repair of a DSB *via* the HR pathway and may be dispensable in chromosomal NHEJ and cells lacking *DMA1* and *DMA2* are hyperefficient in NHEJ plasmid repair. Consistent with their role in HR, we also found that Dma1 and Dma2 may play a role in meiosis, as cells lacking both Dma1 and Dma2 are defective in sporulation (data not shown). Although existing literature and the main body of our work suggest that Dma1 and Dma2 are redundant in function, we were surprised to find that cells lacking DMA2 were significantly less efficient in HR compared with the WT, whereas cells lacking DMA1 showed no statistically significant defect. Overall, these findings indicate that, although the roles of Dma1 and Dma2 are functionally redundant in most respects, future investigation will be necessary to tease apart any possible differential roles for these proteins. In addition, we show that *DMA1* and *DMA2* genetically interact with *RAD9*, which encodes the adaptor protein Rad9, responsible for recruiting Rad53 to the site of damage (69, 70). The human homolog of budding yeast *RAD9*, 53BP1, has been suggested to tip the balance of repair pathway choice in favor of NHEJ (68, 71, 72). This observation in combination with the demonstrated hyperefficient NHEJ phenotype of *dma1Δdma2Δ* suggest that Dma1 and Dma2 may be important for repair pathway selection.

Although Dma1 and Dma2 have been shown to play an important role in degradation of Swe1, regulation of cell cycle, G1 cyclin degradation, and septin dynamics (45, 49, 73, 74, 75), their role on chromatin has never been addressed. Here, we show that Dma1 and Dma2 interact with histones and Rad53 and are localized to gene bodies in a manner similar to the established localization of RNAPII and various other components of transcription machinery (76), indicating that Dma1 and Dma2 may play a role in maintenance of genome stability during transcription elongation. Furthermore, Dma1 and Dma2 are present in low levels on gene bodies in the absence of damage raising the possibility of its role in transcription-coupled genome maintenance. Our results also show that Dma1 and Dma2 are required for proper histone acetylation dynamics during DNA damage, as indicated by attenuated levels of H4 acetylation in the *DMA1* and *DMA2* mutants compared with the WT. The inability to induce histone acetylation may impact the recruitment of key repair proteins or result in the defective eviction of histones upon damage, which impinges on the initiation of recombination-based repair (77, 78, 79).

Although we have established a catalysis-dependent role for Dma1 and Dma2 in the control of genome stability, identification of substrates that are targeted for degradation by these E3 ligases will be important to determine in the future. The human homolog of Dma1 and Dma2, RNF8/RNF168, is known to ubiquitylate H2A.X to assemble the signaling cascade across a DSB site and facilitate the recruitment of repair factors such as 53BP1 (80, 81). Interestingly, RNF8 is also known to regulate the stability of 53BP1 in a DNA damage–dependent manner (82, 83). We showed that *DMA1* and *DMA2* interact genetically with Rad9 (yeast homolog of 53BP1), suggesting the possibility that Rad9 might be a potential target of these proteins. Finally, a recent report showed extensive histone degradation upon DNA damage and its requirement in the repair of DSB *via* the HR pathway (65). Given that Dma1 and Dma2 are required for HR repair and they interact with histones, we speculate that histones could also be a target of Dma1 and Dma2. While we observed a slight degradation of histones upon DNA damage (data not shown), there were no significant differences in the degradation patterns between the WT and the *DMA1* and *DMA2* deletion strains. However, it is quite possible that Dma1 and Dma2 may be controlling degradation of histones locally around DSBs, which will form a significant portion of future investigations. Overall, we have established a role for Dma1 and Dma2 in the regulation of genome stability and DNA damage repair pathway choice; dysregulation of these pathways is an important hallmark of many human diseases including cancer.

## Experimental procedures

### Yeast strains, genotoxic drugs, and antibodies

Yeast strains were grown under standard conditions, and gene deletion and tagging were performed as described previously (84). The list of yeast strains and plasmids used for various studies is provided in Table S1 and S2, respectively. Plasmid transformations were performed as described previously (85). Primers used for deletion and tagging genes are listed in Table S3. Genomic integration of the tagged and the deleted genes was confirmed by PCR. Antibodies used were as follows: anti-HA antibody, ChIP grade (ab9110, Abcam), anti-histone H4 antibody, ChIP grade (ab7311, Abcam), anti-histone H4Ac pan-acetyl antibody, ChIP grade (39243, Active Motif), H2A (1:5000; 39325, Active Motif), H2ASer129Ph (39271, Active Motif), anti-Rad53 (ab104232, Abcam), G6PDH (1:100,000; A9521, Sigma-Aldrich), and rabbit (Amersham NA934; donkey anti-rabbit) and mouse (Amersham NA931; sheep anti-mouse) secondary antibodies were used at 1:10,000.

### Yeast spotting assays

Strains were grown overnight at 30 °C in either yeast extract–peptone–dextrose (YPD; 2% dextrose) nonselective media or media lacking the specific amino acids to maintain plasmid selection. Overnight cultures were diluted to an optical density corresponding to absorbance 0.2 at 595 nm and grown into early-log phase (to an absorbance at 595 nm of about ∼1). Cells were then diluted to absorbance at 595 nm = 0.2, and 5-fold serial dilutions were plated onto the YPD medium (strains containing plasmids were plated onto synthetic complete [SC] media lacking histidine to maintain plasmid selection) containing indicated genotoxic drugs at indicated concentrations.

### NHEJ plasmid repair assay

NHEJ repair assays were performed as described previously (64). Briefly, the pRS416 (*URA* marked) plasmid was linearized by the restriction enzyme digestion with BamHI, producing linear DNA. Recircularization of the plasmid by NHEJ and further maintenance in an episomal form leads to colony formation on plates lacking uracil, which is an indication of repair efficiency (see schematic in Fig. 5*A*). W303 yeast cells were then transformed with linear DNA and plated on SC media lacking uracil. Colonies were counted 3 to 4 days after transformation. Transformation efficiency was controlled for by transforming circular pRS416 into each strain and colonies counted as described. All experiments were performed in biological triplicates, and the colonies were counted manually. Unpaired two-tailed *t* test for means was performed for each experimental sample compared with its respective WT. Alpha was 0.05 in all cases, and all *p*-values are reported in Table S4.

### HR repair assay

HR repair assays were performed as described previously (65). Briefly, the pRS306 plasmid (an integrating plasmid lacking the centromere and autonomously replicating sequence) was linearized by restriction enzyme digestion with StuI at the URA3 gene and was integrated at the URA locus (see schematic in Fig. 5*C*). After transformation, yeast cells were plated on SC media lacking uracil. All experiments were performed in biological triplicates, and the colonies were counted manually. Unpaired two-tailed *t* test for means was performed for each experimental sample compared with its respective WT. Alpha was 0.05 in all cases, and all *p*-values are reported in Table S4.

### Immunoblotting

Asynchronously growing overnight saturated yeast cells were inoculated at an absorbance at 595 nm of 0.2 and allowed to grow until they reached an absorbance at 595 nm of 1.0. Two hundred fifty micrograms per milliliter phleomycin was added to cells when they reached an absorbance at 595 nm of ∼1.0. Trichloroacetic acid extraction of proteins was performed as described previously, and the protein concentration was estimated by using the Bradford assay (86) according to manufacturer’s recommendations. Equal amounts of protein were loaded and run on SDS-PAGE and then transferred to polyvinylidene fluoride membranes. Membranes were incubated with antibodies (as described in the text) to the indicated epitopes. Membranes were incubated with the appropriate secondary antibody conjugated to horseradish peroxidase at 1:10,000 dilution, and blots visualized using enhanced chemiluminescence detection. All experiments were performed in biological triplicates, and the best representative blots are shown.

### Co-IP assays

Co-IP experiments were performed as described previously (87). Briefly, an overnight saturated yeast culture was diluted in 50 ml of YPD to an absorbance at 595 nm of 0.2 and grown subsequently for 4 h to an absorbance at 595 nm of ∼1.0. Cells were washed once with ice-cold water and lysed in 600-μl lysis buffer (450 mM Tris acetate [pH 7.8], 150 mM potassium acetate, 60% [v/v] glycerol, 3 mM EDTA [pH 8.0], supplemented with fresh 1 mM DTT, 1 mM PMSF, 1X complete EDTA-free protease inhibitors [Roche], Universal nuclease, and Benzonase). Lysates were then clarified by centrifugation for 15 min at 4 °C. One milligram of the total protein (estimated using the Bradford assay (86)) was incubated with the indicated antibody overnight at 4 °C in 1 ml of buffer A (50 mM Hepes-KOH [pH 7.5], 1 mM EDTA [pH 8.0], 20% [v/v] glycerol, 125 mM potassium acetate, 1% [v/v] NP-40, supplemented with fresh 100 mM DTT). Protein A Agarose beads (Sigma-Aldrich) were added and incubated for 2 h at 4 °C. The complexes on beads were washed six times in buffer A. Beads were boiled, and proteins were separated by SDS-PAGE and subjected to immunoblotting to detect interacting proteins.

### ChIP

ChIP-qPCR was performed as described previously (88, 89). Briefly, asynchronously growing yeast cells were fixed with 1% formaldehyde and quenched with 0.125 M glycine. After this, cell pellets were lysed using bead beating, and the chromatin was sonicated. One milligram of chromatin was incubated with anti-HA antibody for Dma1 and Dma2 ChIPs. For histone ChIPs, 10 μg of chromatin was incubated with 10 μg of histone H4 and H4(Ac)4 antibody, 1 μg of histone H2A antibody, and 10 μg of H2Aser129-P antibody. Immunoglobulin G and untagged strains were used as negative controls. Data are representative of 2 biological experiments with technical triplicates in each experiment. Statistical analysis to determine the *p*-values was performed using the Prism 8 software.

## Data availability

All the data are contained within the article.

## Supporting information

This article contains supporting information.

## Conflict of interest

The authors declare that they have no conflicts of interest with the contents of this article.
