# Supplementary material for: Catalysis-dependent and redundant roles of Dma1 and Dma2 in maintenance of genome stability in Saccharomyces cerevisiae
Source: J Biol Chem. 2021 Apr 29;296:100721. doi: 10.1016/j.jbc.2021.100721 (PMC8165551; doi:10.1016/j.jbc.2021.100721)
Supplement: Figures S1 and S2 [file mmc1.pdf]

Supplementary Figure 1

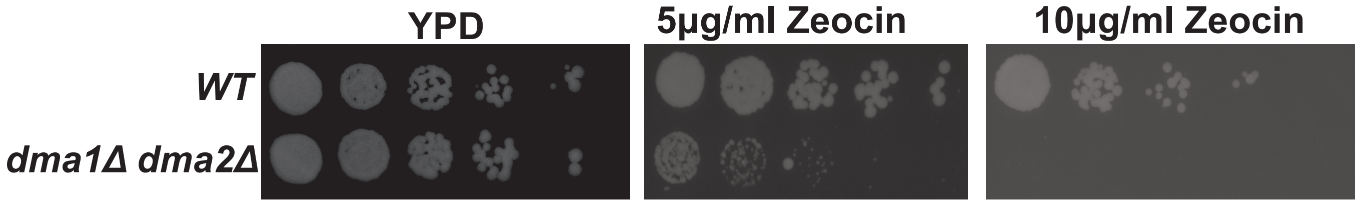

Supplementary Figure 1. Dma1 and Dma2 are required for resistance to Zeocin. Spotting of wild-type and dma1Δdma2Δ strains on indicated concentrations of Zeocin plates.

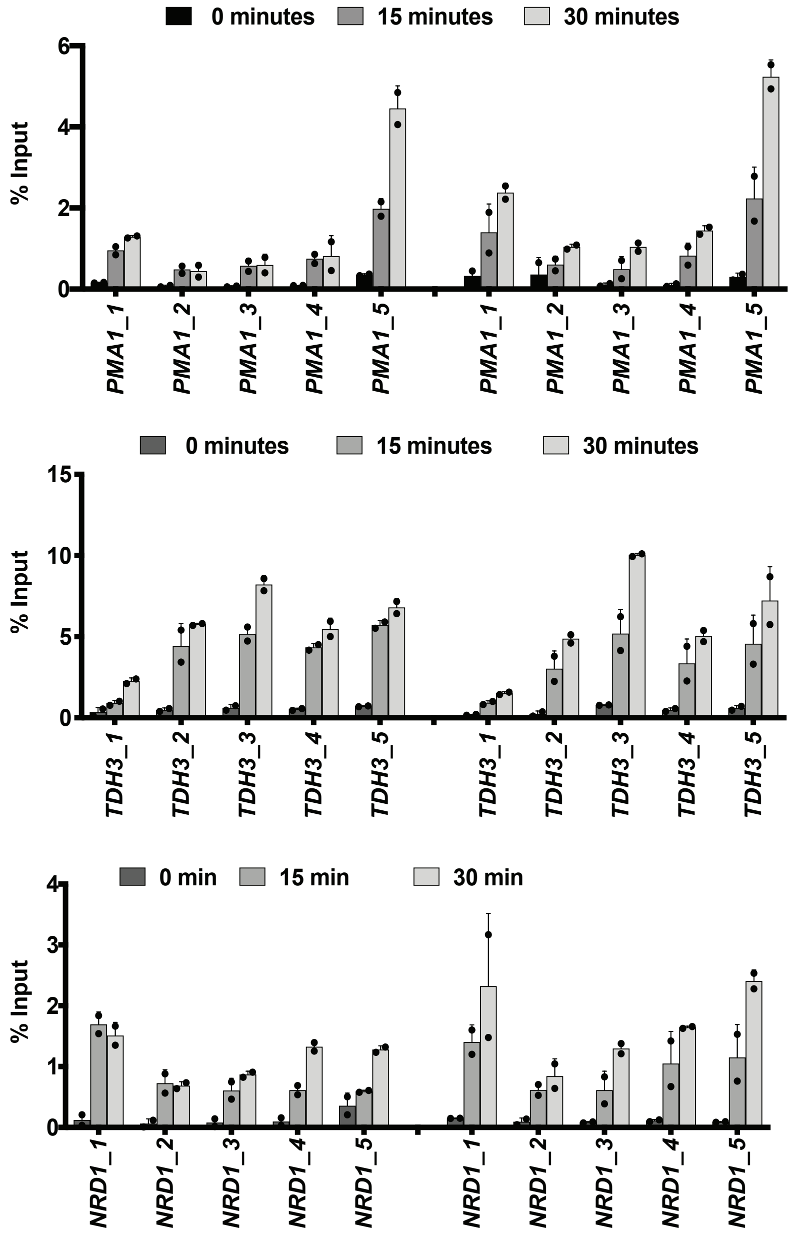

Supplementary Figure 2: Dma1 and Dma2 are not required for the phosphorylation kinetics of histone H2A. Wild-type and *dma1Δdma2Δ* mutant strains were treated with phleomycin (250 µg/mL) under asynchronously growing conditions. Cell were fixed for ChIP as described in materials and methods. ChIP-qPCR showing theincrease in the levels of H2A phosphorylation at ser129 at the candidate loc (A) *PMA1* and (B) *TDH3* nd (C) *NRD1*. % Input is the ratio of p-H2A/H2A at the tested loci.
